# Novel nomograms to predict risk and prognosis in hospitalized patients with severe fever with thrombocytopenia syndrome

**DOI:** 10.3389/fmed.2023.1321490

**Published:** 2023-12-01

**Authors:** Zhenxing Li, Zhaoru Zhang, Chong Chen

**Affiliations:** Department of Infectious Diseases, The Affiliated Chaohu Hospital of Anhui Medical University, Hefei, China

**Keywords:** nomogram, fever, thrombocytopenia syndrome, SFTS virus, *Dabie bandavirus*

## Abstract

**Background:**

Severe fever with thrombocytopenia syndrome (SFTS) is an emerging and life-threatening infectious disease caused by SFTS virus. Although recent studies have reported the use of nomograms based on demographic and laboratory data to predict the prognosis of SFTS, no study has included viral load, which is an important factor that influences the prognosis, when compared with other risk factors. Therefore, this study aimed to develop a model that predicts SFTS prognosis before it reaches the critical illness stage and to compare the predictive ability of groups with and without viral load.

**Methods:**

Two hundred patients with SFTS were enrolled between June 2018 and August 2023. Data were sourced from the first laboratory results at admission, and two nomograms for mortality risk were developed using multivariate logistic regression to identify the risk variables for poor prognosis in these patients. We calculated the area under the receiver operating characteristic curve (AUC) for the two nomograms to assess their discrimination, and predictive abilities were compared using net reclassification improvement (NRI) and integrated discrimination improvement (IDI).

**Results:**

The multivariate logistic regression analysis identified four independent risk factors: age, bleeding manifestations, prolonged activated partial thromboplastin time, and viral load. Based on these factors, a final nomogram predicting mortality risk in patients with SFTS was constructed; in addition, a simplified nomogram was constructed excluding the viral load. The AUC [0.926, 95% confidence interval (CI): 0.882–0.970 vs. 0.882, 95% CI: 35 0.823–0.942], NRI (0.143, 95% CI, 0.036–0.285), and IDI (0.124, 95% CI, 0.061–0.186) were calculated and compared between the two models. The calibration curves of the two models showed excellent concordance, and decision curve analysis was used to quantify the net benefit at different threshold probabilities.

**Conclusion:**

Two critical risk nomograms were developed based on the indicators for early prediction of mortality risk in patients with SFTS, and enhanced predictive accuracy was observed in the model that incorporated the viral load. The models developed will provide frontline clinicians with a convenient tool for early identification of critically ill patients and initiation of a better personalized treatment in a timely manner.

## 1 Introduction

Severe fever with thrombocytopenia syndrome (SFTS), commonly known as “tick disease,” was first reported in China in 2011 (1). It is an emerging infectious disease caused by a new type of bunyavirus carried by ticks [originally named SFTS virus (SFTSV). In 2019, SFTSV was renamed *Dabie bandavirus* and reclassified into the genus *Bandavirus*, family *Phenuiviridae*, and order *Bunyavirales*] (2). Later on, the news of this syndrome reached Korea and Japan (3, 4). Cases of SFTS have also recently been recorded in Vietnam and Myanmar (5, 6), suggesting that the disease has spread more widely. The clinical manifestations of SFTSV include fever, thrombocytopenia, diarrhea, and even circulatory system-wide coagulation and multi-organ dysfunction in severe cases, with a fatality rate ranging from 2.8% to 47% (7, 8). Moreover, according to the World Health Organization’s 2018 annual update of the Blueprint List of Priority Diseases, SFTS is one of the top 10 infectious diseases that demand priority attention (9). Because there is no effective treatment or vaccine to combat this disease, symptomatic and supportive care is essential for patient management, making it necessary for frontline clinicians to recognize patients at risk of life-threatening illness promptly.

It is well known that mathematical modeling of disease progression can help in predicting the success of clinical trials. The nomogram has gained acceptance as a trustworthy statistical tool in recent times. A nomogram is a simple visual graph that uses multiple key parameters to create a statistical model to quantify the risk of a clinical event (10, 11). Nomograms are frequently used in clinical settings to determine and decipher prognostic outcomes in various diseases, such as cancers and coronavirus disease (12–14). Although recent studies have reported the use of nomograms based on demographic and laboratory data to predict the prognosis of SFTS, no study has included viral load, which is more important in influencing the prognosis, compared with other risk factors. This study aimed to develop an accurate and simple model based on viral load and other factors, providing frontline clinicians with a convenient tool for early identification of critically ill patients and initiation of an optimal personalized treatment in a timely manner.

## 2 Materials and methods

### 2.1 Study participants

Between June 2018 and August 2023, 254 patients with SFTS confirmed by laboratory testing who were admitted to the Affiliated Chaohu Hospital of Anhui Medical University were included in this study. The inclusion criteria were as follows: (1) positive serum nucleic acid test and specific viral load and (2) hospitalization for at least 72 h. The exclusion criteria were as follows: (1) age < 18 years; (2) laboratory-confirmed infections with other pathogens, such as Hantaan virus, *Orientia tsutsugamushi*, and rickettsia; (3) history of hematological disorders; and (4) missing clinical data. Finally, 200 patients were enrolled in this study. Because this disease is prevalent in rural areas, many local medical institutions do not have relevant equipment to detect the viral load and have to send samples to the Centers for Disease Control and Prevention or large tertiary hospitals. Therefore, we divided the patients into two groups: with viral load and without viral load. Comparing the detection accuracy of the.

**Figure 1:**
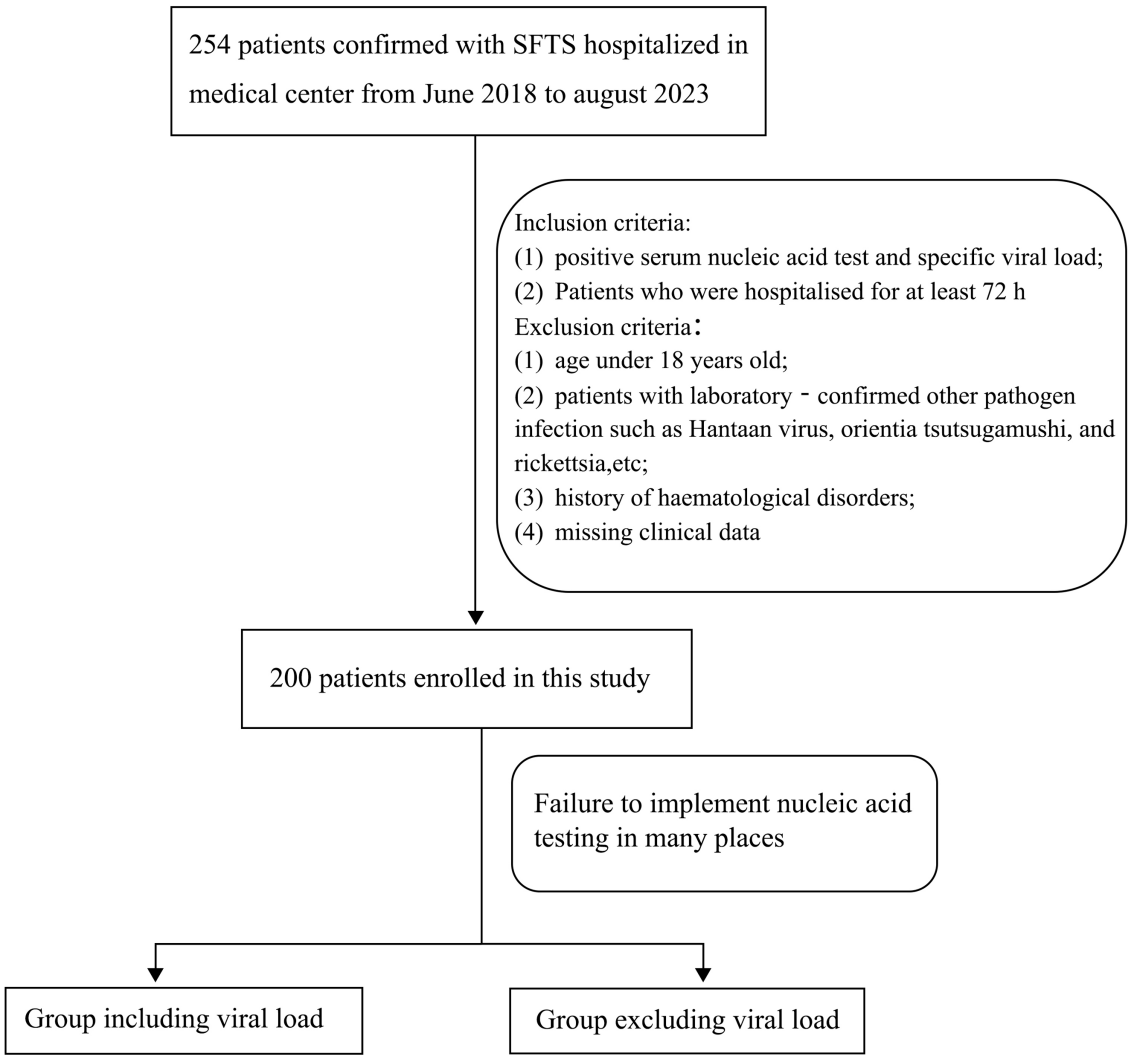
Flow diagram of study profile.

Two models will provide an extra choice and reference for locations where viral RNA load testing for SFTSV is unavailable (Figure 1). The patients were then divided into 165 survivors and 35 non-survivors based on their clinical outcome following admission. The study protocol complied with the Declaration of Helsinki and was approved by the hospital’s ethics review board (No. KYXM-202303-025).

### 2.2 Data gathering

We gathered the patient demographic information (sex, age, illness duration, and outcome), presenting clinical signs, and results of initial post-admission laboratory tests [viral load, routine blood, liver and kidney functions, C-reactive protein (CRP), etc.] from an electronic system. The endpoints observed in this investigation were discharge on better terms or death.

### 2.3 Statistical analysis

Statistical analyses were performed using SPSS version 26 (SPSS Inc., Chicago, IL, United States) and R (version 4.2.2). Comparisons between groups were carried out using two independent samples *t*-tests on measurement data with a normal distribution, which are presented as mean ± standard deviation. Chi-square tests were used to assess categorical variables, which are reported as percentages (*n*, %). Measurement data with a non-normal distribution are presented as medians with interquartile ranges and compared using the Mann–Whitney *U* test. Independent risk factors were identified using univariate and multivariate logistic regression analyses to predict mortality rates. Statistical significance was set at *p* < 0.05.

Two nomograms were developed using independent risk factors for mortality prognosis. We calculated the area under the receiver operating characteristic curve (AUC) of the two nomograms to test model discrimination, and net reclassification improvement (NRI) and integrated discrimination improvement (IDI) were used to assess improvements in risk prediction and measure the usefulness of the new models (15, 16). The calibration curves of the two models indicated excellent concordance, and the net benefit at various threshold probabilities was measured using decision curve analysis.

## 3 Results

### 3.1 Demographic and laboratory indicators

In total, 200 patients met the inclusion criteria (53% female), 17.5% of whom died during hospitalization. The median age of all patients was 71 (range, 59–76) years, and the average time from symptom onset to hospitalization was 4 (range, 3–5) days. The majority of the patients lived in rural areas (78%) and were farmers (76%). Approximately one-quarter (32.5%) had a clear history of tick bites, and less than half (46.5%) had underlying diseases such as hypertension, coronary heart disease, or chronic hepatitis at the time of admission. A high incidence of the SFTS was observed between April and October of each year. The patients were stratified into mortality and survival groups, with only age being significantly different between these groups (*p* < 0.05). Among all patients, 97.5% had fever, 66% fatigue, 74% anorexia, and 41% nausea or vomiting. Eleven clinical symptoms and indicators were compared between the survival and mortality groups. According to these findings, the incidence of bleeding manifestations was significantly higher in the mortality group than in the survival group [18/35 (51.4%) vs. 20/165 (12.1%), *p* < 0.001]. In contrast, the incidence of consciousness disorders was significantly higher in the mortality group than in the survival group [17/35 (48.6%) vs. 32/165 (19.4%), *p* < 0.001]. Bleeding manifestations included skin petechiae, as well as oral, gastrointestinal, and pulmonary bleeding. Consciousness disorders included drowsiness, blurred consciousness, and severe impairment of consciousness (no Glasgow Coma Score examination was performed). Fever, exhaustion, anorexia, and any other symptoms did not significantly differ between the two groups (*p* > 0.05) (Table 1).

**Table 1:** Comparison of demographics and clinical characteristics between the two groups.

Variables	Total	Fatal		*p*-value
		No	Yes	
No.	200	165	35	
Age (years)	71(59–76)	70 (57–75)	76 (73–79)	<0.001
Days of onset (days)	4 (3–5)	4 (3–5)	4 (3–5)	0.332
Female (%)	106 (53)	89 (53.9)	17 (48.6)	0.563
Smoking (%)	32 (16)	24 (14.5)	8 (22.9)	0.223
Alcohol (%)	30 (15)	22 (13.3)	8 (22.9)	0.152
Operation history (%)	33 (16.5)	24 (14.5)	9 (25.7)	0.106
Epidemiology				
Residence, rural/urban, *n* (%)	156/44 (78/22)	126/39 (76.4/23.6)	30/5 (85.7/14.3)	(85.7/14.3) 0.225
Occupations, former/others *n* (%)	152/48 (76/24)	124/41 (75.2/24.8)	28/7 (80/20)	0.542
History of tick bite, *n* (%)	65 (32.5)	55 (33.3)	10 (28.6)	0.585
Underlying diseases, *n* (%)	93 (46.5)	72 (43.6)	21 (60)	0.078
Clinical symptoms, *n* (%)				
Fever	195 (97.5)	160 (97.0)	35 (100)	0.297
Fatigue	132 (66)	107 (64.8)	25 (71.4)	0.455
Anorexia	148 (74)	119 (72.1)	29 (82.9)	0.188
Nausea/vomiting	82 (41)	65 (39.4)	17 (48.6)	0.316
Myalgia	100 (50)	83 (50.3)	17 (48.6)	0.852
Diarrhea	80 (40)	64 (38.8)	16 (45.7)	0.447
Dizzy	35 (17.5)	29 (17.6)	6 (17.1)	0.951
Headache	30 (15)	23 (13.9)	7 (20)	0.362
Enlargement of lymph nodes	50 (25)	43 (26.1)	7 (20)	0.452
Bleeding manifestations	38 (19)	20 (12.1)	18 (51.4)	<0.001
Disturbance of consciousness	49 (24.5)	32 (19.4)	17 (48.6)	<0.001

### 3.2 Laboratory parameters

Most patients had leukopenia, thrombocytopenia, elevated liver enzyme levels, and myocardial impairment. Lymphocyte count, platelet count, and albumin level were significantly lower; levels of aspartate aminotransferase, creatine kinase (CK), CK-MB isoenzyme (CK-MB), lactate dehydrogenase, blood urea nitrogen, serum creatinine, uric acid, activated partial thromboplastin time (APTT), D-dimer, CRP, procalcitonin, and viral load were significantly higher; and prothrombin time (PT) was longer but within the normal range, in the mortality group than in the survival group, while the other biological indicators showed no differences between the two groups (Table 2).

**Table 2:** Comparison of laboratory test indicators between the two study groups.

Variables	Total	Fatal		*p*-value
		No	Yes	
No.	200	165	35	
Leukocytes, × 10^9^/L	2.36 (1.68–3.20)	2.36 (1.68–3.24)	2.39 (1.84–3.00)	0.953
Neutrophils, × 10^9^/L	1.49 (1.04–2.00)	1.44 (1.00–2.04)	1.76 (1.18–1.98)	0.177
Lymphocytes, × 10^9^/L	0.60 (0.44–0.83)	0.62 (0.45–0.86)	0.50 (0.39–0.64)	0.016
Monocytes, × 10^9^/L	0.15 (0.09–0.25)	0.16 (0.10–0.25)	0.12 (0.08–0.26)	0.417
Hemoglobin (g/L)	126.67 ± 17.92	126.90 ± 17.89	125.57 ± 18.23	0.602
Platelets, × 10^9^/L	60.75 ± 19.79	62.81 ± 19.92	51.03 ± 16.16	0.01
ALT (U/L)	54.0 (33.3–99.8)	52.0 (32.5–96.5)	64.0 (40.0–116.0)	0.242
AST (U/L)	135.5 (74.3–280.8)	122.0 (68.0–270.5)	208.0(143.0–512.0)	<0.001
GGT (U/L)	24.0 (16.0–40.8)	23.0 (15.5–38.5)	32.0 (19.0–45.0)	0.055
CK (U/L)	351 (147–844)	279 (142–644)	730 (226–1464)	0.002
CK-MB (U/L)	15.0 (6.4–30.0)	14.0 (5.0–25.1)	25.0 (15.0–51.8)	<0.001
LDH (U/L)	485 (313–853)	469.0 (311.5–822.5)	581 (423–1223)	0.0396
BUN (mmol/L)	7.0 (5.1–9.6)	6.7 (5.0–9.0)	10.0 (7.5–14.5)	<0.001
Creatinine (μmol/L)	77.0 (63.0–100.0)	74.0 (61.0–93.0)	100.0 (88.0–131.0)	<0.001
Uric acid (μmol/L)	275.5 (213.0–359.8)	269.0 (211.5–347.0)	347.0 (251.0–477.0)	0.001
PT (second)	11.83 ± 1.25	11.74 ± 1.26	12.24 ± 1.09	0.04
APTT (second)	45.36 ± 9.40	44.06 ± 8.87	51.53 ± 9.49	<0.001
Fibrinogen (g/L)	2.59 ± 0.62	2.60 ± 0.63	2.52 ± 0.58	0.726
D-dimer	2.71 (1.57–5.41)	2.38 (1.45–4.86)	4.85 (2.74–10.89)	0.001
Potassium (mmol/L)	3.74 ± 0.51	3.72 ± 0.52	3.83 ± 0.49	0.161
Sodium (mmol/L)	133.58 ± 3.91	133.58 ± 3.89	133.56 ± 4.09	0.776
Calcium (mmol/L)	1.98 ± 0.13	1.98 ± 0.13	1.95 ± 0.14	0.124
Albumin (g/L)	34.94 ± 4.28	35.44 ± 4.20	32.54 ± 3.89	<0.001
CRP (mg/L)	2.67 (0.67–7.42)	2.35 (0.50–5.32)	7.60 (3.26–9.56)	<0.001
PCT (ng/mL)	0.23 (0.11–0.54)	0.20 (0.11–0.44)	0.66 (0.29–2.27)	<0.001
Viral load (lg copy/mL)	4.77 (3.83–5.91)	4.50 (3.63–5.57)	6.15 (5.38–6.75)	<0.001

ALT, alanine transaminase; APTT, activated partial thromboplastin time; AST, aspartate aminotransferase; CRP, C-reactive protein; CK, creatine kinase; CK-MB, creatine kinase-MB isoenzyme; GGT, gamma-glutamyl transferase; LDH, lactate dehydrogenase; BUN, blood urea nitrogen; PCT, procalcitonin; PT, prothrombin time.

### 3.3 Screening of predictors

**Figure 2:**
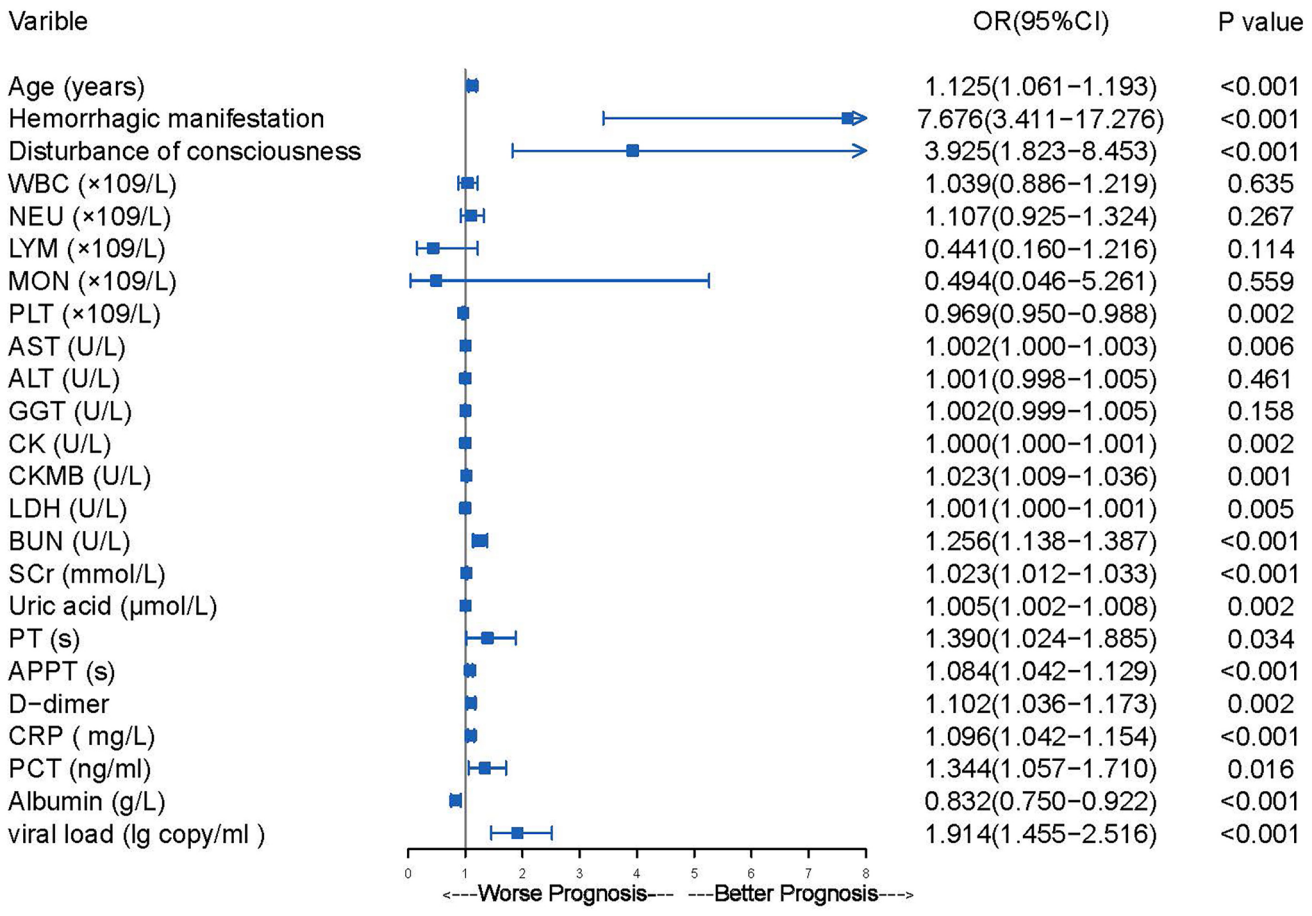
The results of univariate logistic regression analysis of mortality risk in 200 patients hospitalized with SFTS.

Univariable logistic regression analysis revealed that older age; hemorrhagic manifestation; disturbance of consciousness; reduced platelet count; elevated levels of aspartate aminotransferase, CK, CK-MB, lactate dehydrogenase, blood urea nitrogen, serum creatinine, uric acid, PT, APTT, D-dimer, CRP, procalcitonin, albumin, and viral load were risk factors for mortality (Figure 2). Multivariable logistic regression analysis was performed to identify variables that could predict critical illness in patients with SFTS (Table 3). In the group with viral load, the variables associated with poor prognosis were older age, hemorrhagic manifestations, prolonged APTT, and viral load (*p* ≤ 0.001 for all) (Table 3). In the group without viral load, the variables related to poor prognosis were older age, hemorrhagic manifestations, and prolonged APTT (*p* < 0.001 for all) (Table 3).

**Table 3:** Multivariable logistic regression analysis of the mortality risk in 200 patients with viral load.

(A) Group with viral load			
Variable	Coefficient (*B*)	OR (95% CI)	*p*-value
Age (years)	0.142	1.152 (1.058–1.255)	0.001
Hemorrhagic manifestations (Yes vs. No)	2.524	12.474 (3.894–39.955)	<0.001
APPT (s)	0.152	1.164 (1.088–1.246)	<0.001
Viral load (lg copy/mL)	0.778	2.178 (1.494–3.176)	<0.001

(B) Group without viral load			
Variable	Coefficient (*B*)	OR (95% CI)	*p*-value
Age (years)	0.140	1.150 (1.064–1.242)	<0.001
Hemorrhagic manifestations (Yes vs. No)	2.205	9.073 (3.259–25.260)	<0.001
APPT (s)	0.133	1.142 (1.079–1.208)	<0.001

APTT, activated partial thromboplastin time; OR, odds ratio; CI, confidence interval.

**Figure 3:**
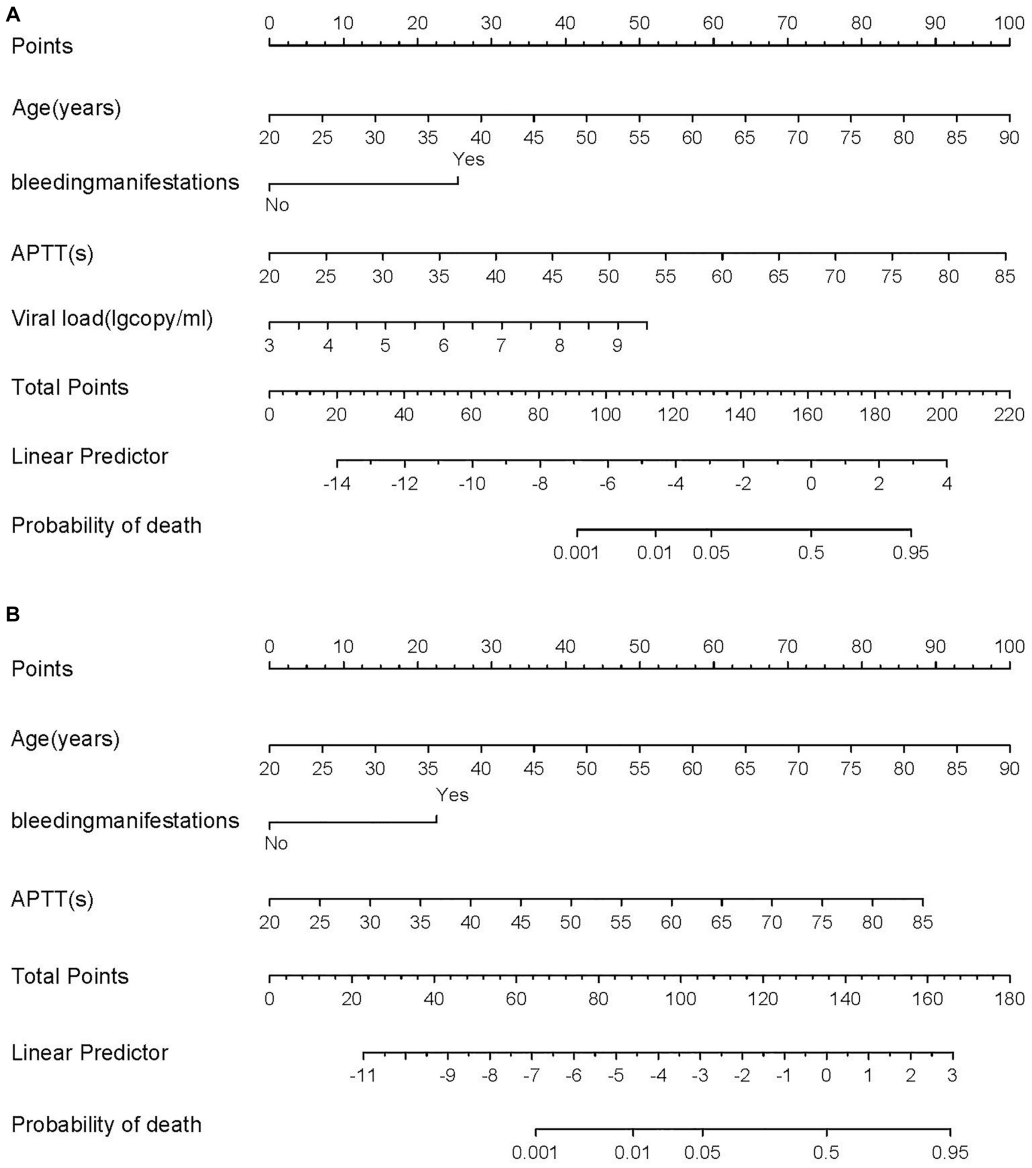
**(A)** The nomogram for predicting risk of death with SFTS. **(B)** The brief nomogram for predictor risk of death with SFTS.

**Figure 4:**
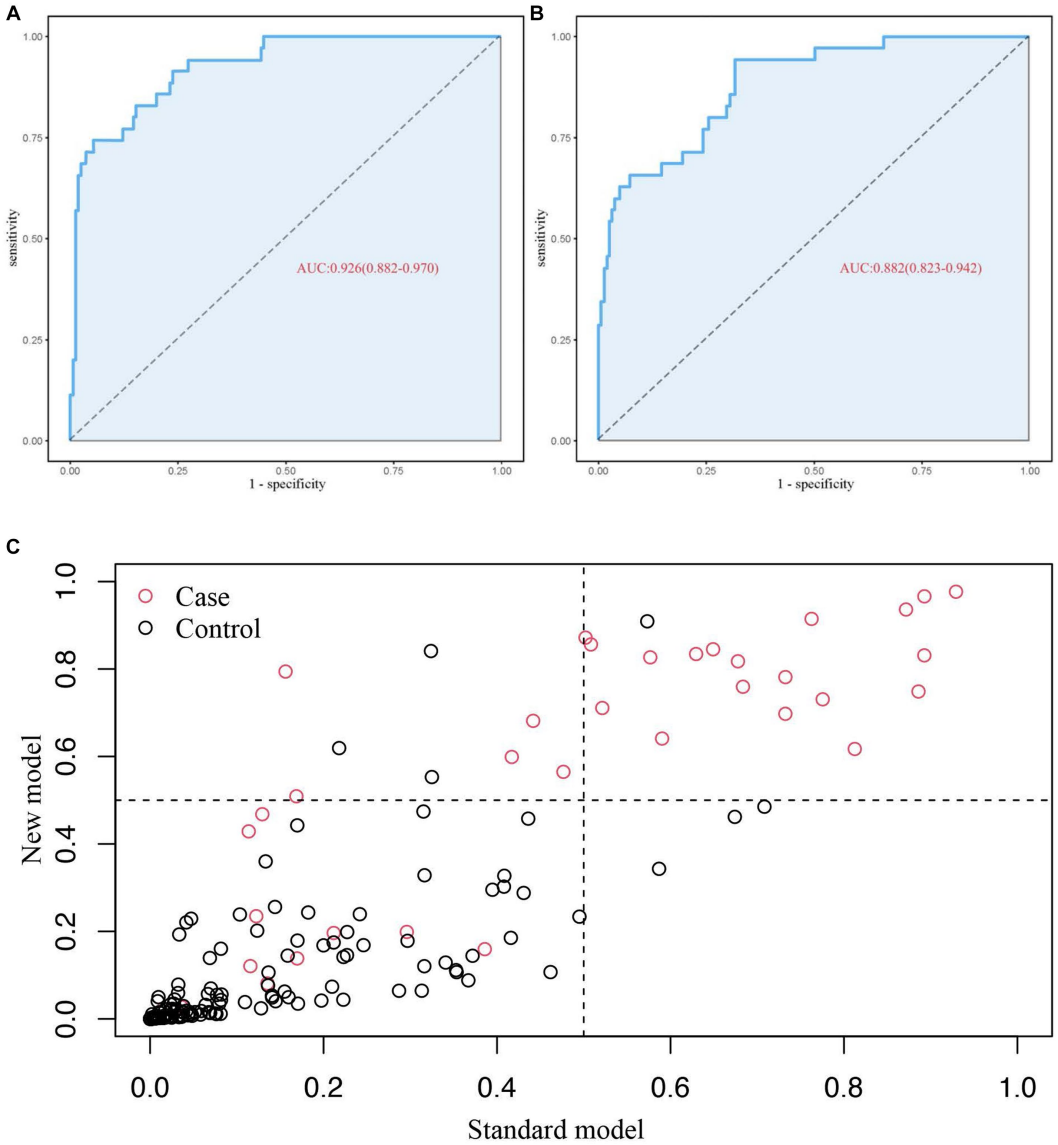
**(A)** ROC curve of 4 predictors, **(B)** ROC curve of 3 predictors, **(C)** the result graph NRI.

**Figure 5:**
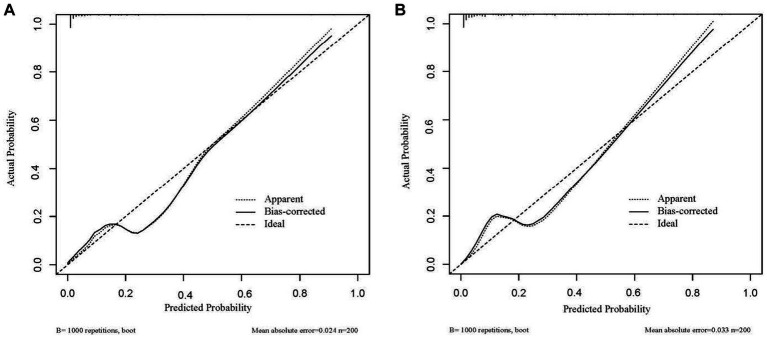
**(A)** The calibration curve of 4 predictors, **(B)** the calibration curve of 3 predictors.

**Figure 6:**
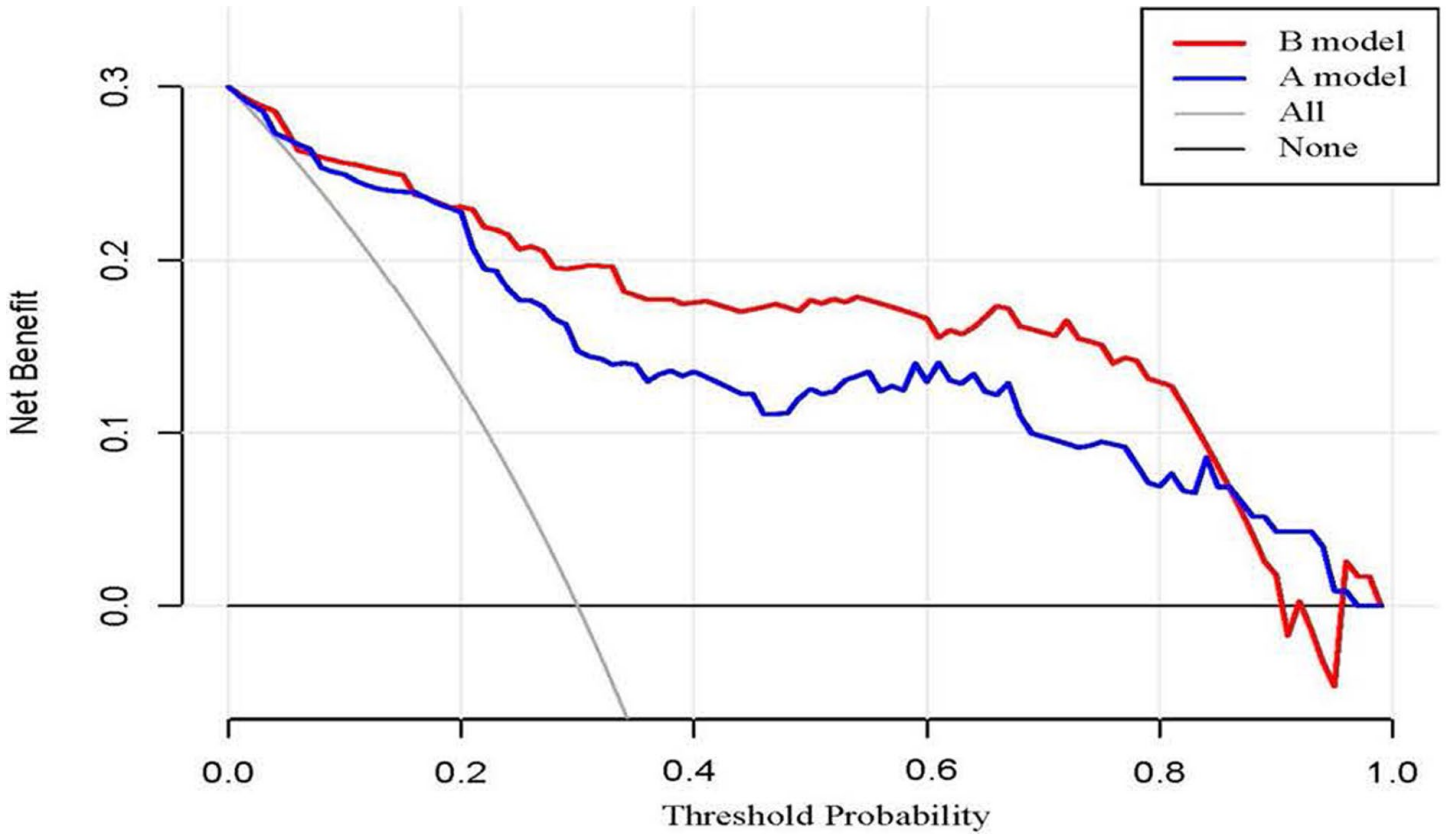
**(A)** DCA of 4 predictors, **(B)** DCA of 3 predictors.

Based on the four factors identified in the group with viral load, a final nomogram predicting mortality risk in patients with SFTS was constructed (Figure 3A); another brief nomogram excluding the viral load was also constructed (Figure 3B). The AUC [0.926, 95% confidence interval (CI): 0.882–0.970 vs. 0.882, 95% CI: 0.823–0.942] and NRI (0.143, 95% CI: 0.036–0.285) were calculated and compared between the two models (Figure 4). In addition, we also calculated IDI (0.124, 95% CI, 0.061–0.186) between the two models. These results suggest that the former model is more accurate than the latter for predicting patient prognosis. Both calibration plots showed remarkable concordance between the predicted probability of mortality and actual observations, with mean absolute errors of 0.024 and 0.033, respectively (Figure 5). The decision curve analysis showed a potential net benefit with rather broad threshold probability distributions (Figure 6).

## 4 Discussion

SFTS is an emerging infectious disease caused by the *Dabie bandavirus* with a rapid progression and high fatality rate. Early identification of patients with poor prognosis is particularly important (8). Consequently, we conducted a retrospective examination of demographic data, clinical manifestations and symptoms, laboratory results, and indicators of mortality risk in 200 patients with SFTS. Since this disease is prevalent in rural areas and the cost of SFTSV testing equipment is high, many local healthcare facilities do not perform this test and thus, SFTSV results are not available in a timely manner. Therefore, we divided the patients into two groups based on whether the viral load was included and plotted nomograms. We aimed to compare the difference between the two groups regarding poor prognosis of SFTS and, at the same time, provide a reference for those who are unable to obtain viral load immediately. This study is the first to incorporate viral load into a model to predict the potential for poor prognosis in patients with SFTS, and better predictive accuracy was observed in the model that incorporated viral load (AUC: 0.926 vs. 0.882; NRI 0.143, 95% CI: 0.036–0.285; IDI 0.124, 95% CI: 0.061–0.186). According to Kwon et al. (17), there is a substantial difference in plasma viral RNA levels between survivors and non-survivors upon admission. Hayasaka et al. (18) performed a molecular imaging of SFTSV-induced infectious diseases in A129 mice infected with a lethal dose of the virus. The findings of this study support the idea that high viral load is a strong risk factor for catastrophic results in patients with SFTS. This may be related to the fact that high viral load induces higher levels of IFN-inducible protein-10 and macrophage inflammatory protein-1 while blocking the release of activated normal T cell-expressed and secreted factors, even more leading to severities or even death (19). Nevertheless, this investigation also indicated that advanced age was a significant contributor to SFTS mortality, consistent with previous reports. Qian et al. (20) constructed a risk model to forecast fatalities in patients with SFTS based on three high-risk variables: age, APTT, and CRP to lymphocyte ratio. According to research by Jung et al. (21), older age was linked to a higher 30 days mortality rate in patients with SFTS (adjusted hazard ratio: 1.10; 95% CI:1.04–1.17). This may be related to the prevalence of underlying disorders, reduced immunity, and higher incidence of morbidity and death in many older individuals. In addition, in ferrets with anatomical and physiological characteristics similar to those of humans, older ferrets infected with SFTSV were found to exhibit more severe clinical signs and higher mortality rates, than younger ferrets (22, 23). The three stages of the clinical course of SFTS are fever, multi-organ dysfunction, and convalescence. Jia et al. (24) discovered that APTT and thrombin time in patients that died were noticeably longer throughout the fever and multi-organ dysfunction stages and that APTT had high sensitivity and specificity in predicting mortality (84.85% and 81.65%, respectively). Song et al. (25) demonstrated a link between fatal outcomes in patients with SFTS and coma, pulmonary infection, high viral load, and prolonged APTT. In addition, Xu et al. (26) observed that prolonged APTT and bleeding manifestations were early independent warning factors for mortality, which is in line with our study. Additionally, we noticed that APTT was significantly prolonged in the mortality group (*p* < 0.001), whereas PT was significantly different between the mortality and survival groups (*p* = 0.034); however, PT was within the normal range and not significantly prolonged. This may be related to the absence of coagulation factor XI, which Mizoe et al. (27) demonstrated to be the likely cause of APTT prolongation in SFTS. If APTT prolongation is triggered by coagulation factor deficits, plasma-derived or recombinant coagulation factors may serve as alternatives for the management of bleeding tendency (28, 29).

In line with the literature, our study revealed that bleeding symptoms were more frequent in the mortality group than in the survival group. This was the most significant risk factor of the parameters, both in the group with and without viral load [odds ratio (OR) 1 = 12.474; 95% CI: 3.894–39.955, OR 2 = 9.073; 95% CI: 3.259–25.260, respectively]. Li claimed that the emergence of hemorrhagic symptoms (adjusted OR = 2.79; *p* < 0.001) was a risk factor for SFTS mortality. The possible mechanisms of hemorrhagic signs as a risk factor for mortality may be thrombocytopenia and endothelial dysfunction (30). Patients with SFTS who experience severe thrombocytopenia may see a reduction in thrombin synthesis; in addition, SFTSV increases vascular permeability by destroying vascular endothelial cells, which are capable of causing extensive skin ecchymosis, as well as tissue and organ hemorrhages in patients with SFTS (31, 32). Most importantly, practically all bleeding manifestations had a strong causal relationship with mortality, indicating the importance of regularly monitoring bleeding symptoms over the course of the illness.

This investigation established nomograms based on older age, presence of bleeding manifestations, prolonged APTT, and viral load. The models indicated the likelihood of critical illness in patients with SFTS, and our internal validation supported the model’s efficacy. Analyzing risk factors can help predict severe illness, enabling proper care, and maximizing the use of available medical resources.

However, there are three limitations associated with the study design. First, because this was a single-center retrospective study, the quality and generalizability of the data may have been affected. Second, as APTT prolongation is a risk factor for predicting the prognosis of SFTS, SFTS in combination with other conditions that may cause APTT prolongation were not considered, such as antiphospholipid antibody syndrome, etc. Third, the models were not externally validated. For this reason, we are undertaking a prospective study in a larger cohort of patients with SFTS admitted to other healthcare facilities and after August 2023 to validate the predictive value of the model.

## 5 Conclusion

This study identified several risk variables for SFTS, including advanced age, presence of bleeding symptoms, prolonged APTT, and viral load. Two critical risk nomograms were developed based on these indicators for the early prediction of mortality risk in patients with SFTS, and enhanced predictive accuracy was observed in the model that incorporated viral load. The findings of this study may be of great significance for clinical applications.

## Data availability statement

The original contributions presented in the study are included in the article/supplementary material, further inquiries can be directed to the corresponding author.

## Ethics statement

The studies involving humans were approved by the Ethics Committee of the Affiliated Chaohu Hospital of Anhui Medical University (No. KYXM-202303-025). The studies were conducted in accordance with the local legislation and institutional requirements. The participants provided their written informed consent to participate in this study. Written informed consent was obtained from the individual(s) for the publication of any potentially identifiable images or data included in this article.

## Author contributions

ZL: Writing – original draft, Writing– review & editing. ZZ: Writing – original draft, Writing – review & editing. CC: Data curation, Investigation, Writing – original draft.
